# Effects of Dietary Indole-3-carboxaldehyde Supplementation on Growth Performance, Intestinal Epithelial Function, and Intestinal Microbial Composition in Weaned Piglets

**DOI:** 10.3389/fnut.2022.896815

**Published:** 2022-05-16

**Authors:** Ruofan Zhang, Guowen Huang, Yuting Ren, Haifeng Wang, Yanxin Ye, Jiaqing Guo, Mengting Wang, Weiyun Zhu, Kaifan Yu

**Affiliations:** ^1^Laboratory of Gastrointestinal Microbiology, Jiangsu Key Laboratory of Gastrointestinal Nutrition and Animal Health, College of Animal Science and Technology, Nanjing Agricultural University, Nanjing, China; ^2^National Center for International Research on Animal Gut Nutrition, Nanjing Agricultural University, Nanjing, China; ^3^Changjia Agricultural Technology Co., Ltd., Shanghai, China

**Keywords:** Indole-3-carboxaldehyde, intestinal epithelial proliferation, intestinal barrier function, gut microbiota, weaned piglets

## Abstract

As a microbial tryptophan metabolite, indole-3-carboxaldehyde (ICA) has been suggested to confer benefits to host, such as regulation of intestinal barrier function. This study aimed to elucidate the role of ICA in modulating intestinal homeostasis via using a weaned pig model. Twenty-four weaned piglets were randomly allocated into three groups: the control group (a basal diet), ICA100 group (the basal diet supplemented with 100 mg/kg ICA), and ICA200 group (the basal diet supplemented with 200 mg/kg ICA). The experiment lasted 14 d, and pigs from the control and ICA100 groups were slaughtered. The results showed no significant differences in the average daily gain (ADG) and average daily feed intake (ADFI) among the three groups (*P* > 0.05). However, the ICA100 group had a lower feed to gain ratio (F:G) (*P* < 0.05). Dietary ICA supplementation did not alter the villus height, crypt depth, and villus height/crypt depth ratio in the small intestine, and did not change the intestinal permeability and antioxidant parameters (*P* > 0.05). Intriguingly, ICA treatment significantly increased the jejunal, ileal and colonic indexes in piglets (*P* < 0.05). Besides, the expression of proliferating cell nuclear antigen (PCNA) in the intestine was up-regulated by ICA treatment. Moreover, *in vitro* experiments demonstrated that 15 μM ICA significantly accelerated the proliferation activity of IPEC-J2 cells, and increased the expression of the ICA receptor aryl hydrocarbon receptor (AHR) and the proliferation markers PCNA and Cyclin D1 (*P* < 0.05). In addition, dietary ICA supplementation modulated the intestinal flora, increasing the richness estimators and diversity index, decreasing the abundances of phylum *Fibrobacterota* and genera *Alloprevotella, Prevotella*, and *Parabacteroides*, and enriching the abundance of genera *Butyrivibrio*. These data reveal a beneficial role for the microbial metabolite ICA on intestinal epithelial proliferation, rather than intestinal barrier function, in weaned piglets.

## Introduction

Early weaning of piglets allows sows to improve reproductive efficiency, whereas weaning stress causes a series of problems in piglet health, such as intestinal dysfunction, microbial dysbiosis and systematic inflammation, which leads to diarrhea, poor growth performance, and even death (1, 2). The development of intestinal epithelium and the maintenance of intestinal barrier are crucial to ensure intestinal health and achieve efficient production in pigs. Gut microbes participate in and regulate the intestinal barrier, and the rich and diverse microbial metabolites are important players in the microbe-host interaction. Studies have shown that some specific microbial metabolites affect the development of intestinal epithelium and regulate intestinal barrier. For instance, butyrate was found to increase intestinal cell proliferation in piglets by using a cecal fistula infusion model (3). Lee et al. (4) revealed that microbiota-derived lactate accelerates intestinal stem-cell-mediated epithelial development. Dietary butyrate supplementation modified intestinal barrier function by promoting tight junction protein expression, and attenuated diarrhea in weaned piglets (5). The vital role of microbial metabolites affecting intestinal function has attracted increasing attention.

Indole-3-carboxaldehyde (ICA), an indole derivative produced by bacterial catabolism of tryptophan, has potential effects on intestinal epithelial function. Several studies have indicated that indole enhances intestinal barrier function by up-regulating the expression of tight-junction proteins, and promotes intestinal homeostasis (6–8). Indole-3-propionic acid, another indole derivative, decreased paracellular permeability, enhanced trans-epithelial electrical resistance, and increased tight junction proteins (9) (claudin-1, occludin, and ZO-1). ICA has been reported to provide mucosal protection and ameliorate mouse models of colitis (10). Moreover, Samantha et al. determined that ICA protects against increased gut permeability caused by colitis in mice, and maintains the integrity of the apical junctional complex and its associated actin regulatory proteins (11). Taken together, there is a potential role of the tryptophan-indole derivative ICA in regulating intestinal barrier function and intestinal inflammation. However, it is unknown whether ICA can affect the intestinal integrity and intestinal development of weaned piglets, thereby improving their growth performance.

Accordingly, the present study was performed to explore the effects of dietary ICA supplementation on the growth performance, intestinal integrity, and gut microbiota composition in weaned piglets, aimed to elucidate the role and mechanism of ICA in improving intestinal homeostasis.

## Materials and Methods

### Animals, Housing, and Sampling

Pigs were raised and maintained on an experimental farm in Jiangsu Province, China. All animal care procedures in the experiment were operated according to the Chinese Experimental Animal Care and Use guidelines, and were approved by the Animal Care and Use Committee of Nanjing Agricultural University. A total of twenty-four weaned Landrace barrows, 28 d of age, were randomly allocated into three groups based on body weight: the control group (a basal diet, shown in Table 1), ICA100 group (the basal diet supplemented with 100 mg/kg ICA), and ICA200 group (the basal diet supplemented with 200 mg/kg ICA). The indole-3-carboxaldehyde compound (catalog number: 129445) was purchased from Sigma Inc, MO, USA. Animals were placed into individual pens with free access to feed and water. The body weight, feed intake, and diarrhea scoring were recorded during d 0 to d 14 of the trial. Scores were 0 = normal, firm feces; 1 = soft feces, possible slight diarrhea; 2 = definitely unformed, moderately fluid feces; 3 = very watery and frothy diarrhea. The diarrhea rate and diarrhea index were calculated according to the formulas (12): diarrhea rate (%) = Σ (the number of pigs with diarrhea per pen × days of diarrhea) / (total number of piglets × 14 d) × 100; diarrhea index = sum of diarrhea scores of pigs per pen / (number of piglets per pen × total days). Pigs from the control group and the ICA100 group were slaughtered at d 14. Organs and intestinal segments were isolated and weighed, samples including serum, jejunum, ileum, colon, and colonic digesta were collected. A small segment of jejunum and ileum were fixed in 4% paraformaldehyde for subsequent histological and immunofluorescence analyses. Other samples were stored at−80°C for further analyses.

**Table 1:** Ingredients and nutrient composition of the basal diet.

**Ingredients**	**Content, %**	**Nutrient level**	**Content, %**
Extruded corn	24.00	Net energy, Kcal/kg	2530
Rice broken	16.81	Crude protein	18.00
Flour	5.00	Ether extract	5.24
Fermented soybean meal	8.00	Crude fiber	1.38
Extruded soybean	5.00	Ash	5.32
Fish meal	3.00	Calcium	0.76
Soy protein concentrate	4.00	Available phosphorus	0.45
Milk powder	10.00	SD-lysine	1.50
Whey powder (low protein)	10.00	SD-methionine	0.60
Sucrose	3.00	SD-cysteine	0.22
Glucose	4.00	SD-(M+C)	0.82
High nucleotide yeast hydrolysate	1.00	SD-The	0.90
Soybean oil	0.65	SD-tryptophan	0.26
Limestone	0.80		
Ca(H_2_PO_4_)_2_	0.74		
Premix^1^	4.00		
Total	100.00		

*^1^Provided per kg of premix: vitamin A, 32,000 KIU; vitamin D3, 8,000 KIU; vitamin E, 200,000 mg; vitamin K3, 10,000 mg; vitamin B1, 10,094 mg; vitamin B2, 20,000 mg; vitamin B6, 12,054 mg; vitamin B12, 80 mg; folic acid, 5,985 mg; D-calcium pantothenate, 99,960 mg; biotin, 1,000 mg; niacinamide, 50,490 mg; Cu (copper sulfate pentahydrate), 10,000 mg; Fe (as ferrous sulfate monohydrate), 50,100 mg; Zn (as zinc sulfate), 32,205 mg; Mn (manganese), 14,628 mg; Se (seleniwm yeast), 150 mg; I (calcium iodate), 85 mg; Cr (chromium picolinate), 78 mg*.

### Cell Culture

The intestinal porcine epithelial cell (IPEC-J2) line was used in this study. IPEC-J2 cells were cultured in Dulbecco's Modified Eagle Medium/Nutrient Mixture F-12 (DMEM/F12; #L310KJ, Shanghai Basalmedia Technologies Co., Ltd., Shanghai, China) supplemented with 10% fetal bovine serum (FBS). Cells were incubated at 37°C in a mixture of 5% carbon dioxide and 95% air. The cell culture medium was changed every 2 days. Indole-3-carboxaldehyde (Sigma Inc, MO, USA) was dissolved in DMSO and then diluted with DMEM/F12 medium to prepare the ICA treatment solution. IPEC-J2 cells were treated with 0, 1, 2, 5, 10, 15, and 20 μM ICA, respectively. After 24 h of ICA treatment, the cell viability and proliferation ability were detected, and the cells were collected to analyze the expression of cell proliferation-related proteins.

### Cell Viability and Proliferation Assays

Cell viability and proliferation ability were measured by using cell counting kit 8 (CCK8) assay and 5-ethynyl-2'-deoxyuridine (EdU) incorporation assay (#C0075S; BeyoClick™ EdU Cell Proliferation Kit with Alexa Fluor 555) according to the manufacturer's instructions, respectively. Briefly, cells were seeded into 96-well-culture plates, and exposed to different doses of ICA solutions for 24 h. CCK8 reagent was added to each well, and the absorbance was evaluated by a microplate reader at 450 nm wavelength. Cell viability was expressed by cell survival rate, which was calculated according to the provided formula. EdU working fluid was added to each well, and fluorescence staining was observed under a Zeiss confocal laser microscope. The average intensity of fluorescence images was evaluated by ImageJ 1.53 m. Cell proliferation ability was assessed by the ratio of EdU-positive cells to 4′,6-diamidino-2-phenylindole (DAPI) cells per well.

### Serum Antioxidant Capacity

Serum antioxidant capacity was reflected by Total superoxide dismutase (T-SOD), glutathione peroxidase (GSH-Px), catalase (CAT), Total antioxidant capacity (T-AOC), and Malondialdehyde (MDA) activities. These indicators were determined in serum by using colorimetric kits purchased from Nanjing Jiancheng Institute of Bioengineering following the manufacturer's protocol.

### D-lactic Acid, Diamine Oxidase, and Endotoxin

Serum D-lactic acid content, diamine oxidase (DAO) activity, and endotoxin content was analyzed using commercial ELISA kits (Nanjing Jiancheng Institute of Bioengineering, Nanjing, China) of Porcine D-lactic acid, Porcine diamine oxidase, and Porcine endotoxin, respectively. All procedures were performed according to the manufacturer's instructions. The absorbance values were detected by the enzyme labeling analyzer (Rayto RT-6100, Shenzhen Rayto Life Science Co., Ltd, Guangdong, China).

### Histological and Immunofluorescence Analyses

The intestine samples were stained with hematoxylin and eosin (H&E) for histological analysis, and stained with immunofluorescence for statistics of target protein expression and distribution. After fixation in 4% paraformaldehyde, the intestine tissue was dehydrated, embedded in paraffin wax, and sectioned at 4 μm. Tissue sections were dewaxed in xylene and rehydrated to water through a series of graded ethanol, then stained with hematoxylin and eosin, and sealed with neutral gum. The morphology of the intestine was observed by a microscope (Nikon ECLIPSE BOi, Japan). The villus height and crypt depth were measured by the Nis-Elements 4.20 software (Nikon, ECLIPSE BOi, Japan). Briefly, the immunofluorescence staining process of jejunum tissue was mainly dewaxing to water, antigen retrieval, circle drawing, serum blocking, first antibody incubation, second antibody incubation, DAPI counterstaining the nucleus, autofluorescence quenching, and mounting. Finally, the slices were photographed with a confocal laser scanning microscope (Zeiss LSM 900 META, Jena, Germany), and images were processed with the ZEN software (Zeiss).

### Real-Time Quantitative PCR

Total RNA was extracted from the jejunum using an RNAeasy kit (Aidlab Bio CO.Ltd, Beijing, China) following the manufacturer's protocols. PrimerScript TM RT Reagent kit (TaKaRa, Japan) was used to convert RNA to cDNA. QPCR assays were performed on the Step One Plus real-time PCR System using SYBR Premix Ex Taq dye (TaKaRa, Japan). Primer sequences are listed: *claudin-1* (F: GAGGATGGTCACACCGTGGT, R: GGAGGATGCTGTTGTCTCGG), *occludin* (F: ATGCTTTCTCAGCCAGCGTA, R: AAGGTTCCATAGCCTCGGTC), *ZO-1* (zonula occludens 1) (F: ACAGGAGGGAAGCCATTTTCA, R: ATTTAAGGACCGCCCTCTCC), β*-actin* (F: AGAGCGCAAGTACTCCGTGT, R: ACATCTGCTGGAAGGTGGAC).

### DNA Isolation and 16s rRNA Gene Sequencing

Total bacterial DNA was extracted from the colonic digesta using a bead-beating and phenol-chloroform method as previously described by Zoetendal et al. (13). DNA concentration was measured by a Nano-Drop 1000 spectrophotometer (Thermo Scientific Inc, Wilmington, DE, USA). The extracted DNA samples were sent to Biozeron Biotechnology Co., Ltd (Shanghai, China) for 16s rRNA gene sequencing. The V3-V4 regions of the bacterial 16S rRNA gene were amplified by PCR using universal primers 341F (5′-CCTAYGGGRBGCASCAG-3′) and 806R (5′-GGACTACNNGGGTATCTAAT-3′). The Qubit 3.0 fluorometer was used to quantify each PCR product for uniform mixing. The Illumina Truseq DNA Library Construction Kit was used for the library construction of mixed samples. Sequencing was conducted on an Illumina platform.

### Western Blot Analysis

Total protein of IPEC-J2 cells were extracted with RIPA protein lysate (Beyotime Technology, Shanghai, China) containing 1% protease inhibitor. Protein concentrations were measured by a bicinchoninic acid (BCA) protein assay kit (#A045-4-2, Nanjing Jiancheng Bioengineering Institute, Jiangsu, China). Equivalent amounts of protein with 4 × loading buffer were denatured at 98°C for 10 min, followed by cooling on ice. The denatured protein was separated by 12% SDS-PAGE gel and transferred to the PVDF membrane (Millipore, Bedford, MA, USA). Then, the PVDF membrane was blocked with 5% skim milk for 1 h at room temperature, and incubated with the primary antibodies PCNA (#GB11010, Servicebio Technology Co., Ltd., Wuhan, China), Cyclin-D1 (#GB111372, Servicebio Technology Co., Ltd.), AHR (#A00225-4, BOSTER Biological Technology Co., Ltd.), and β-actin (#20536-1-AP, Proteintech Group, Inc., IL, USA) at 4°C overnight. After being washed three times with TBST, the PVDF membrane was incubated with the secondary antibody for 1 h at room temperature. Similarly, the PVDF membrane was washed three times with TBST. Finally, the target protein bands were visualized by using a chemiluminescence system (Tanon, Shanghai, China). Band intensities were quantified using ImageJ 1.53 m software, and all results were expressed as target protein/β-actin protein ratio.

### Statistical Analysis

All statistical analyses were performed using Statistical Product and Service Solutions (version 26.0, SPSS, Inc., IBM, Chicago, IL, USA). Figures were generated using GraphPad Prism 8 software (San Diego, CA, USA). Data are presented as mean ± standard error of the mean (SEM), and compared using one-way analysis of variance (ANOVA) for multiple comparisons and independent samples *t*-test for two-group comparisons. All differences were considered significant at *P* < 0.05.

## Results

### Growth Performance

The effects of dietary ICA treatment on ADG, ADFI and F:G are presented in Table 2. There were no significant differences in ADG and ADFI among the three groups (*P* > 0.05). However, compared with the control group, the ICA100 had a lower F:G during the experimental period (*P* < 0.05). In addition, no significant difference was observed in the diarrhea rate and diarrhea index among dietary treatments (*P* > 0.05).

**Table 2:** Effects of dietary ICA supplementation on the growth performance of weaned piglets^1^.

**Items^**2**^**	**Control**	**ICA100**	**ICA200**	***P*-value**
Initial BW, kg	9.31 ± 0.43	9.00 ± 0.39	9.04 ± 0.44	0.861
Final BW, kg	14.54 ± 0.76	14.84 ± 0.53	14.10 ± 0.78	0.752
ADG, g/d	402.75 ± 30.65	449.45 ± 24.68	388.56 ± 34.44	0.363
ADFI, g/d	656.10 ± 27.81	588.91 ± 46.33	565.56 ± 34.42	0.223
F:G	1.98 ± 0.20^a^	1.48 ± 0.05^b^	1.82 ± 0.12^ab^	0.049
Diarrhea rate, %	2.91 ± 0.68	2.93 ± 1.06	2.81 ± 1.52	0.997
Diarrhea index	0.28 ± 0.09	0.32 ± 0.11	0.31 ± 0.19	0.982

^1^*Data are presented as means with standard errors, n = 8 per treatment. Different superscript letters in the same row indicate significant differences between groups (P < 0.05). ICA, indole-3-carboxaldehyde; ICA100, the basal diet supplemented with 100 mg/kg ICA; ICA200, the basal diet supplemented with 200 mg/kg ICA*.

*^2^BW, body weight; ADG, average daily gain; ADFI, average daily feed intake; F:G, feed to gain ratio*.

*Different superscript letters in the same row indicate significant differences between groups (P < 0.05) explains the meaning of the letter “a,b”*.

### Organ Indexes

**Figure 1:**
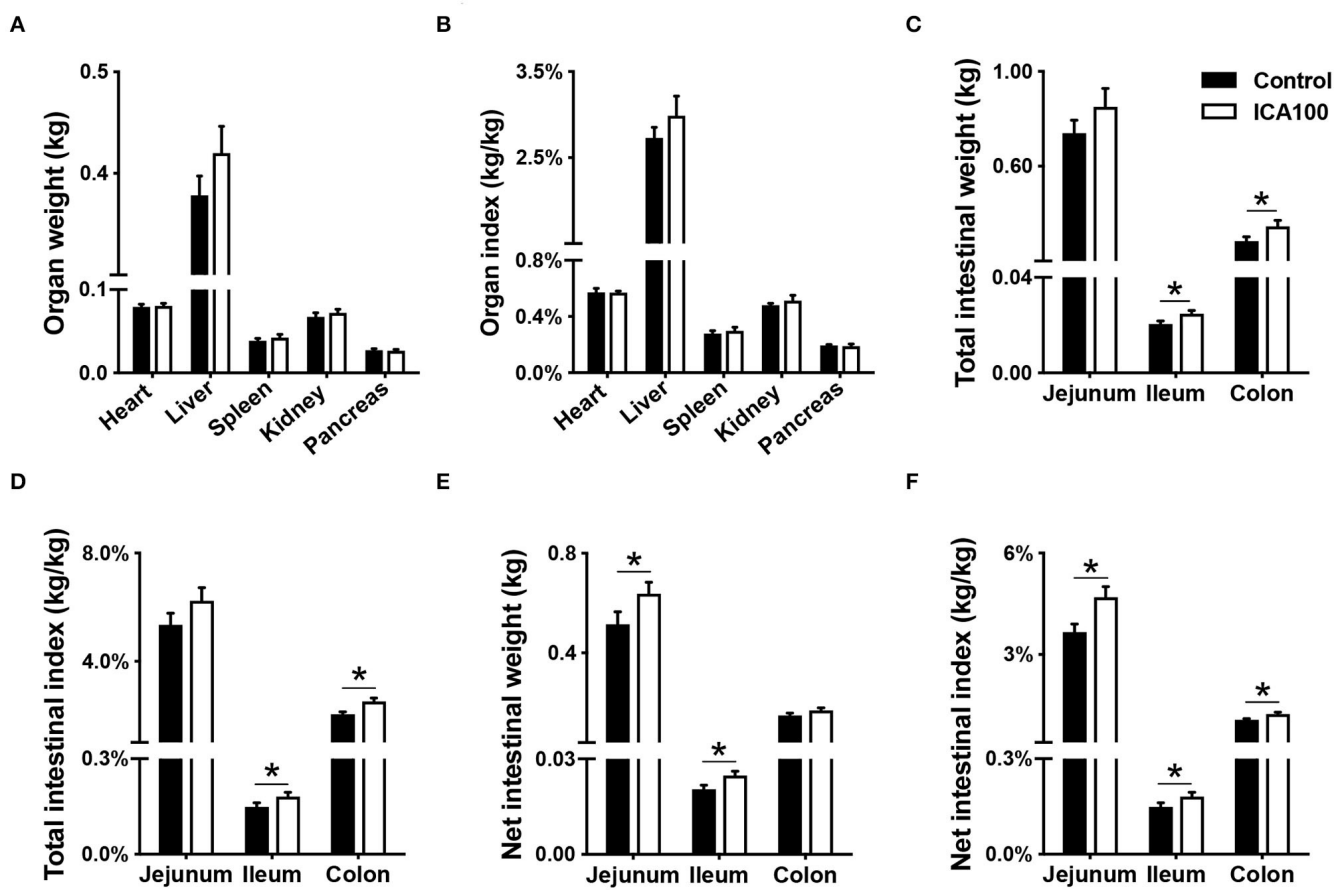
Effects of dietary ICA supplementation on organ indexes of weaned piglets. The weight **(A)** and organ index **(B)** of the heart, liver, spleen, kidney and pancreas in the control and ICA100 groups. The total intestinal weight **(C)**, total intestinal index **(D)**, net intestinal weight **(E)**, and net intestinal index **(F)** in the control and ICA100 groups. Data are expressed as mean ± SEM. *P < 0.05 vs. the Control group. ICA100, the basal diet supplemented with 100 mg/kg indole-3-carboxaldehyde.

The weight and indexes of the major organs, including heart, liver, spleen, kidney, and pancreas, did not differ between the control and ICA100 groups (*P* > 0.05) (Figures 1A,B). Nevertheless, dietary ICA supplementation changed the indexes of digestive organs in piglets. Compared with the control group, the ICA100 group had significantly higher total ileal weight, total ileal and colonic indexes, net ileal index, and net jejunal, ileal and colonic indexes (*P* < 0.05) (Figures 1C–F).

### Intestinal Morphology, Permeability, and Tight-Junction Protein Levels

**Figure 2:**
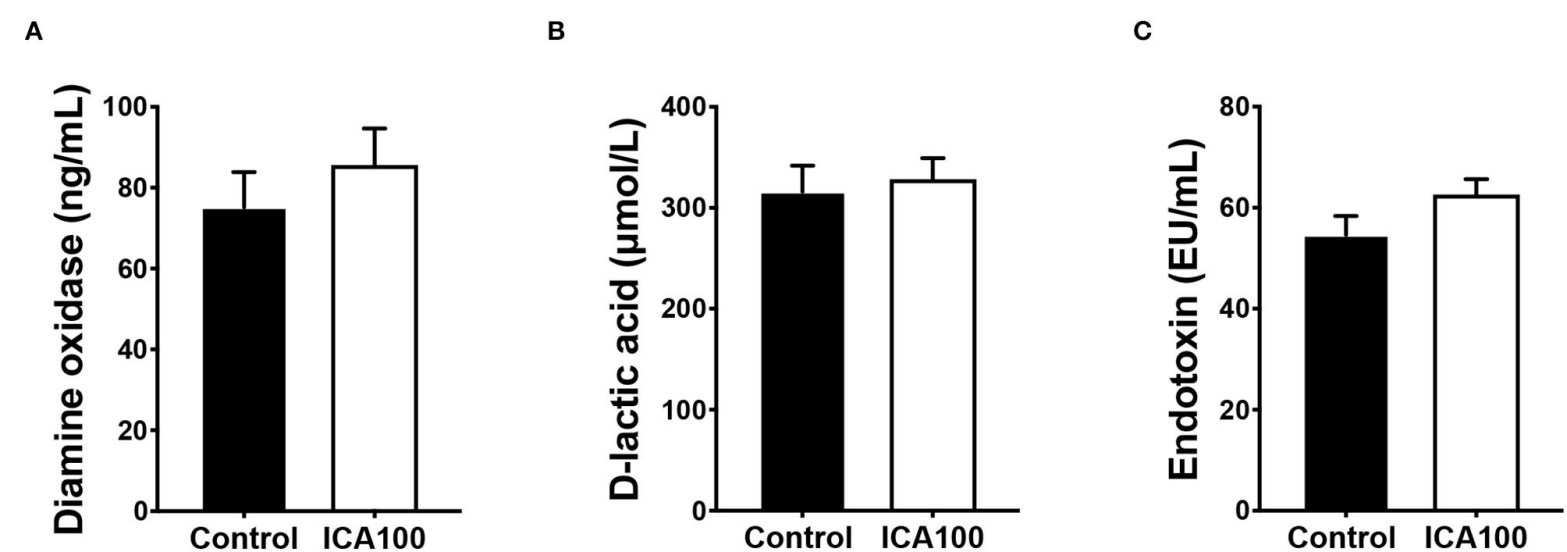
Effects of dietary ICA supplementation on the DAO activity **(A)**, D-lactic acid concentration **(B)**, and endotoxin content **(C)** in serum of weaned piglets. Data are expressed as mean ± SEM. ICA100, the basal diet supplemented with 100 mg/kg indole-3-carboxaldehyde. DAO, diamine oxidase.

**Figure 3:**
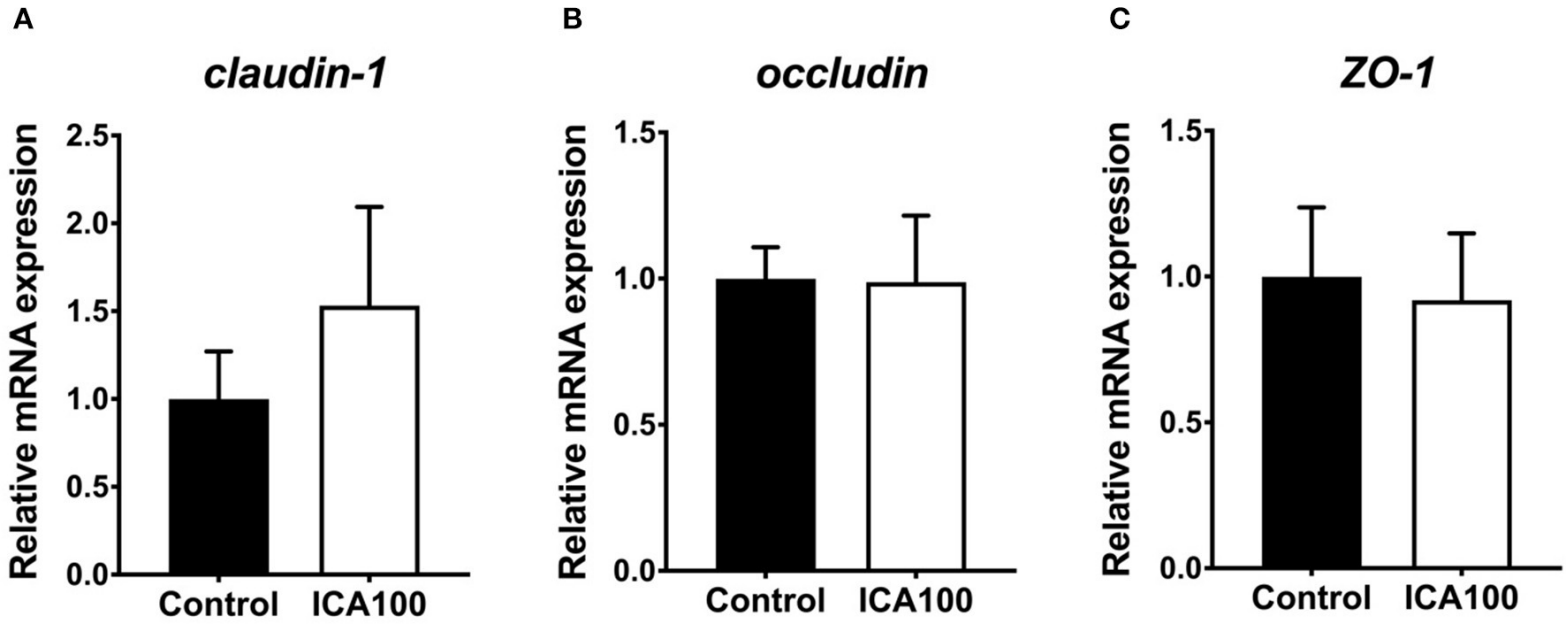
Effects of dietary ICA supplementation on the mRNA expression of *claudin 1* **(A)**, *occludin* **(B)**, and *ZO-1* **(C)** in the jejunum of weaned piglets. Data are expressed as mean ± SEM. ICA100, the basal diet supplemented with 100 mg/kg indole-3-carboxaldehyde. ZO-1, zonula occludens 1.

The intestinal morphological characteristics are shown in Table 3. Dietary ICA supplementation did not alter the villus height, crypt depth, and villus height/crypt depth ratio in the jejunum and ileum of piglets (*P* > 0.05). To evaluate the effect of ICA on intestinal permeability, serum diamine oxidase activity (DAO), D-lactic acid, and endotoxin concentration were measured. No significant difference was observed in these indicators between the control and ICA100 groups (*P* > 0.05) (Figure 2). In addition, the mRNA levels of *claudin-1, occludin*, and *ZO-1* in the jejunum showed no difference between the two groups (*P* > 0.05) (Figure 3).

**Table 3:** Effects of dietary ICA supplementation on the intestinal morphology of weaned piglets^1^.

**Items^**2**^**	**Control**	**ICA100**	***P*-value**
Villus height of jejunum, μm	445.13 ± 29.55	442.90 ± 31.29	0.960
Crypt depth of jejunum, μm	306.62 ± 23.84	345.71 ± 14.54	0.187
VH:CD of jejunum	1.49 ± 0.11	1.29 ± 0.09	0.177
Villus height of ileum, μm	328.50 ± 17.46	302.77 ± 9.24	0.234
Crypt depth of ileum, μm	205.79 ± 13.85	179.20 ± 18.40	0.262
VH:CD of ileum	1.64 ± 0.12	1.80 ± 0.20	0.494

*^1^Data are expressed as mean ± SEM, n = 8 per treatment. ICA100, the basal diet supplemented with 100 mg/kg indole-3-carboxaldehyde*.

*^2^CD, Crypt depth; VH, Villus height*.

### Serum Antioxidant Parameters

**Figure 4:**
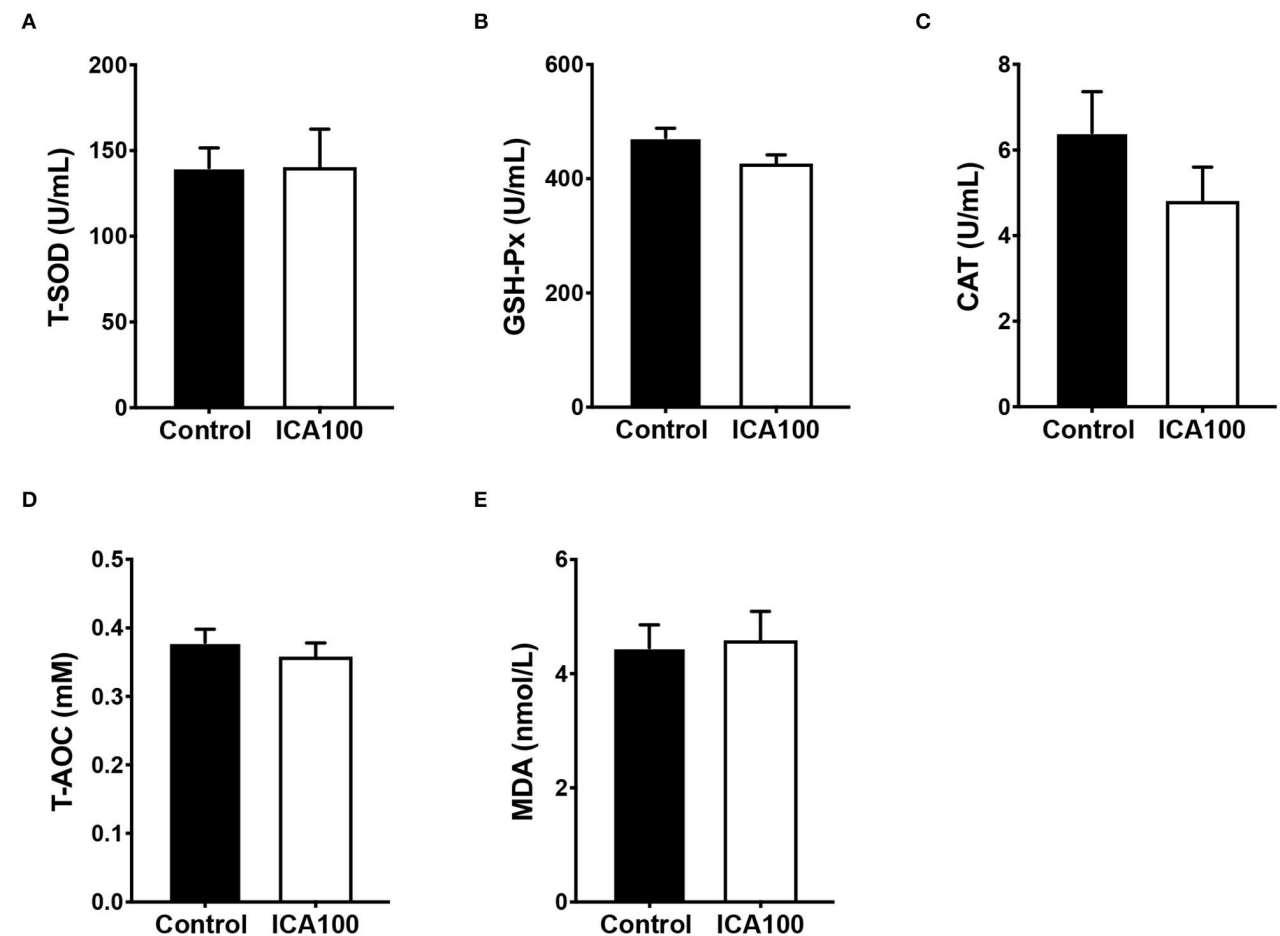
Effects of dietary ICA supplementation on the activities of T-SOD **(A)**, GSH-Px **(B)**, and CAT **(C)**, and the contents of T-AOC **(D)**, and MDA **(E)** in serum of weaned piglets. Data are expressed as mean ± SEM. ICA100, the basal diet supplemented with 100 mg/kg indole-3-carboxaldehyde. T-SOD, total superoxide dismutase; GSH-Px, glutathione peroxidase; CAT, catalase; T-AOC, total antioxidant capacity; MDA, malondialdehyde.

To determine whether ICA affected oxidative stress in weaned piglets, we examined antioxidant enzyme activities in serum. The results showed that the levels of superoxide dismutase (SOD), glutathione peroxidase (GSH-Px), catalase (CAT), total antioxidant capacity (T-AOC), and malondialdehyde (MDA) were no significant differences between the control and ICA100 groups (*P* > 0.05) (Figure 4).

### Intestinal Epithelial Cell Proliferation *in vivo*

**Figure 5:**
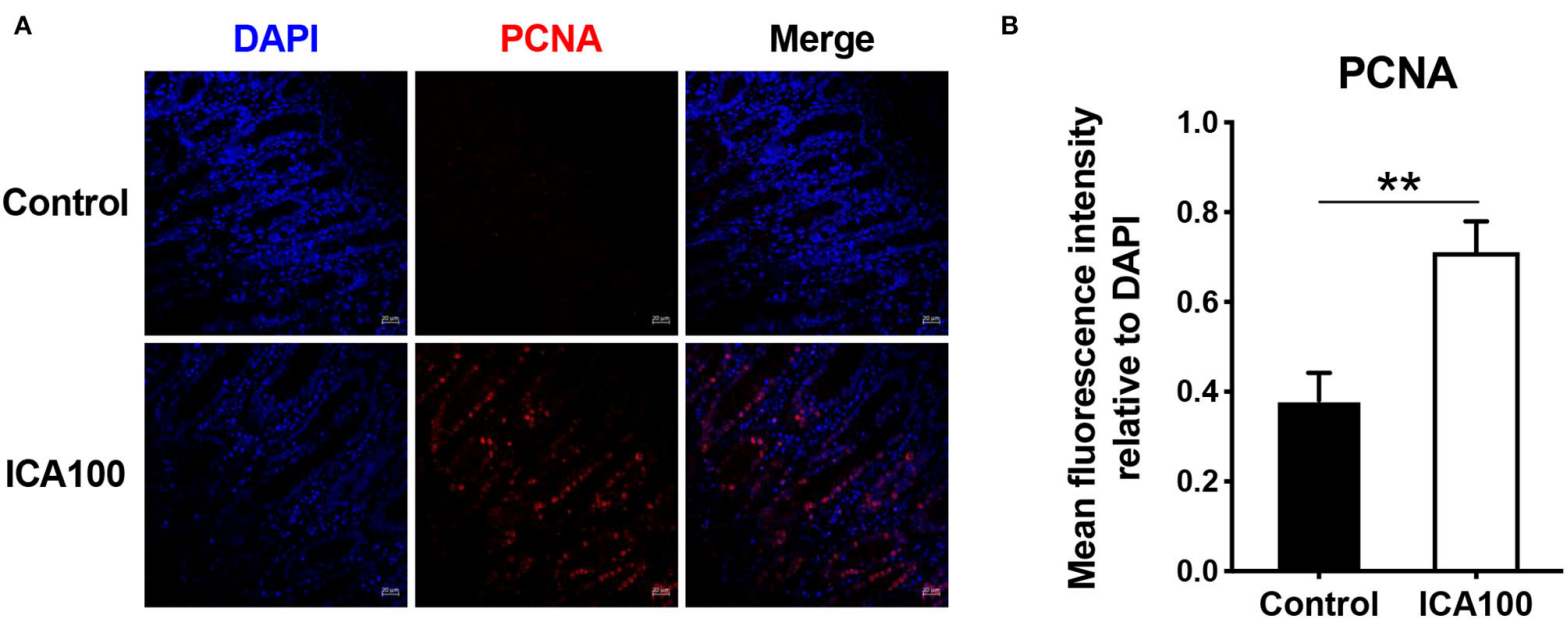
ICA promotes the proliferation of intestinal epithelial cells *in vivo*. The jejuna were stained for PCNA (red), and the nuclei were stained with DAPI (blue). Data are expressed as mean ± SEM. **P < 0.01 vs. the Control group. ICA100, the basal diet supplemented with 100 mg/kg indole-3-carboxaldehyde. PCNA, proliferating cell nuclear antigen.

Since dietary ICA supplementation increased intestinal tissue weight, we analyzed the expression of proliferation marker protein by using immunohistochemical staining. As shown in Figure 5, the jejunal proliferating cell nuclear antigen (PCNA) levels was significantly increased in piglets fed diet with ICA compared to the control group (*P* < 0.05).

### Intestinal Epithelial Cell Proliferation *in vitro*

**Figure 6:**
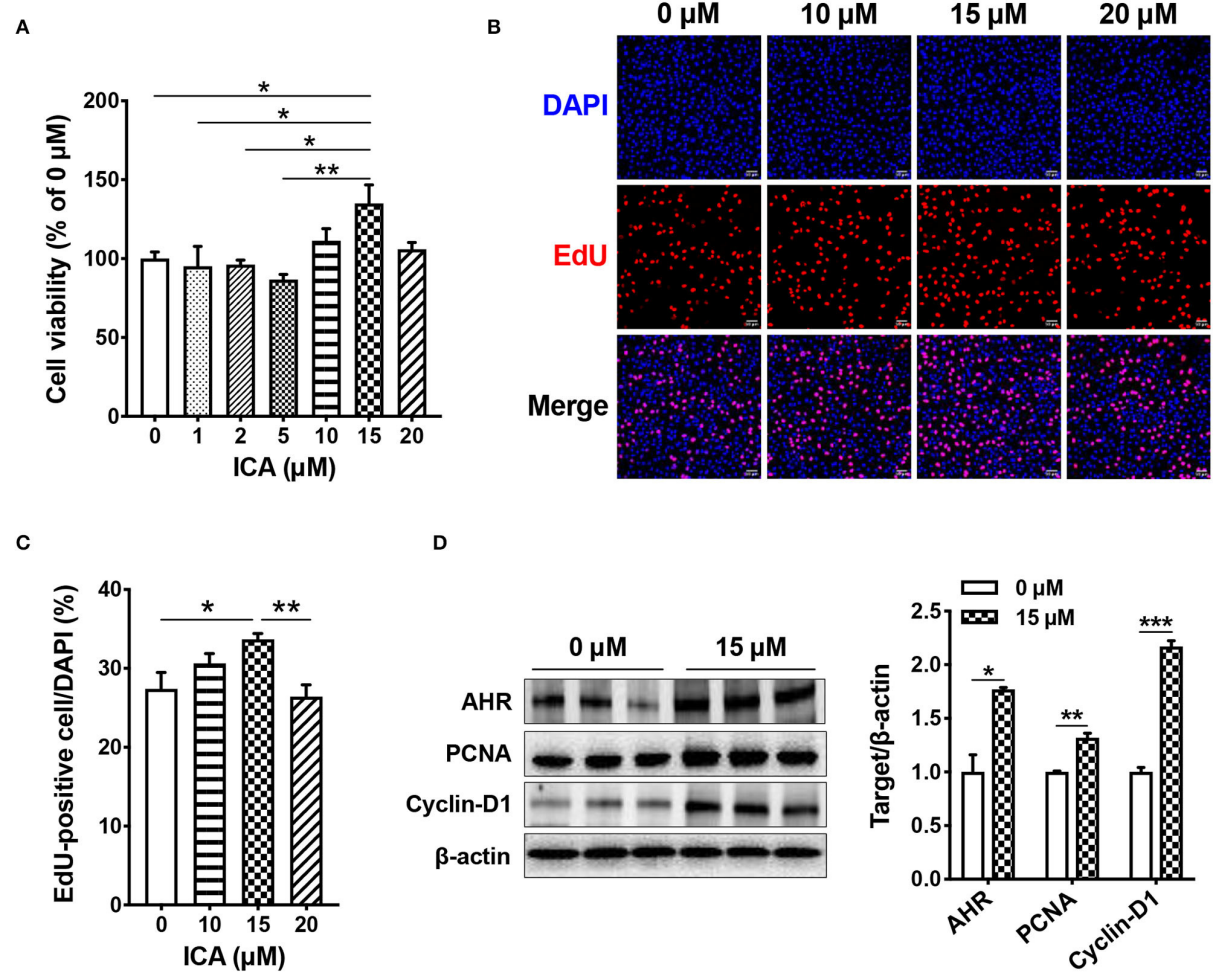
ICA accelerates the proliferation of intestinal epithelial cells *in vitro*. IPEC-J2 cells were treated with different doses of ICA for 24 h. The cell viability was determined by CCK8 assay **(A)**. The cell proliferation was determined by EdU incorporation assay **(B,C)**. Cells were stained for EdU (red), and the nuclei were stained with DAPI (blue). The expression of AHR, PCNA, and Cyclin D1 were measured by western blot **(D)**. Data are expressed as mean ± SEM. *P < 0.05, **P < 0.01, ***P < 0.001 vs. the Control group. ICA100, the basal diet supplemented with 100 mg/kg indole-3-carboxaldehyde. AHR, aryl hydrocarbon receptor; PCNA, proliferating cell nuclear antigen.

We further assessed the effects of ICA on the proliferation of intestinal epithelial cells in the cell model of IPEC-J2. Cells were treated with 0, 1, 5, 10, 15, and 20 μM ICA for 24 h. ICA at the concentration of 10, 15, and 20 μM promoted cell viability of IPEC-J2, determined by CCK 8 assay, with 15 μM significantly higher than the control (*P* < 0.05) (Figure 6A). Meanwhile, the EdU incorporation assay showed that 15 μM ICA led to a significant increase in the number of EdU^+^ cells in IPEC-J2 cells (*P* < 0.05), indicating accelerated cell proliferation (Figures 6B,C).

Furthermore, we analyzed the expression of aryl hydrocarbon receptor (AHR, the ICA receptor) and the proliferation markers PCNA and Cyclin D1 by using western blot. The results showed that ICA treatment significantly up-regulated their expression in the intestinal epithelial cells (*P* < 0.05) (Figure 6D).

### Gut Microbiota Composition

**Figure 7:**
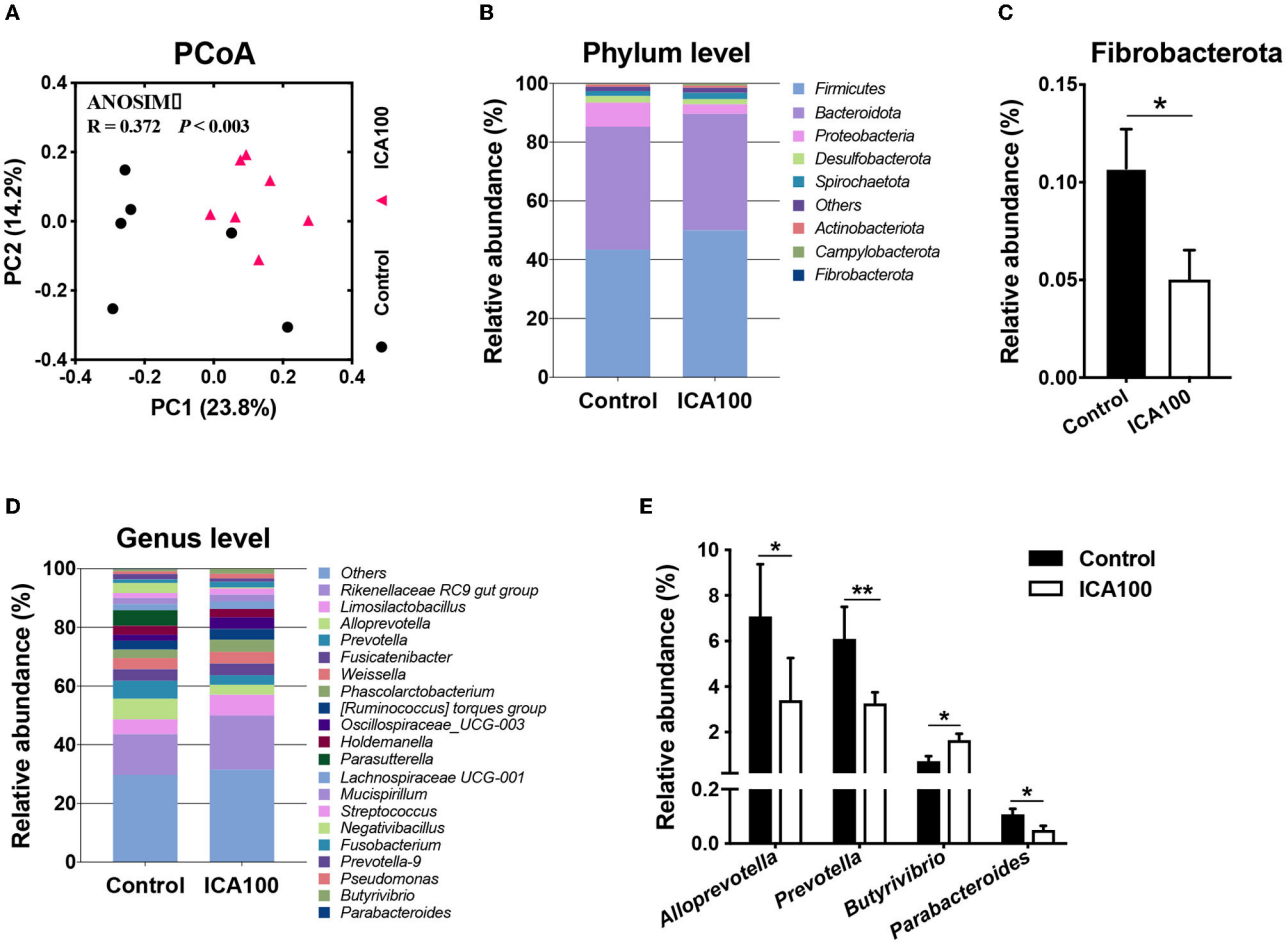
Effects of dietary ICA supplementation on the colonic microbiota composition of weaned piglets. Principal coordinate analysis plot based on the Bray-Curtis index **(A)**. Relative abundance of reads at phylum-level **(B)** and significantly different phyla **(C)**. Relative abundance of reads at genus-level **(D)** and significantly different genera **(E)**. Data are expressed as medians. *P < 0.05, **P < 0.01 vs. the Control group. ICA100, the basal diet supplemented with 100 mg/kg indole-3-carboxaldehyde.

Colonic microbiota composition was evaluated by 16S rRNA gene sequencing (OTUs listed in the Supplementary Material). The results showed that ICA supplementation significantly increased the richness estimators (abundance-based coverage estimator [ACE] and Chao1) (*P* < 0.05) (Table 4). The ICA100 group also had an increased α diversity with a higher Shannon index and a higher Simpson index compared with the control group (*P* < 0.05). Principal-coordinate analysis (PCoA) plots based on Bray-Curtis index showed a significant separation between the two groups with PC1 and PC2 at 23.8% and 14.2% of the explained variance, respectively (Figure 7A). At the phylum level, *Firmicutes* and *Bacteroidota* were the most dominating phyla in the colon from piglets (Figure 7B). The ICA100 group showed a lower relative abundance of the phylum *Fibrobacterota* compared with the control group (*P* < 0.05) (Figure 7C). At the genus level, *Rikenellaceae RC 9 gut group, Limosilactobacillus, Alloprevotella*, and *Prevotella* were the dominant genera (Figure 7D). Dietary ICA supplementation significantly increased the abundance of *Butyrivibrio* (*P* < 0.05). Conversely, ICA treatment reduced the abundances of *Alloprevotella, Prevotella*, and *Parabacteroides* (Figure 7E).

**Table 4:** Alpha diversity of colonic microbial community in weaned piglets^1^.

**Items**	**Control**	**ICA100**	***P*-value**
Chao1	704.97 ± 19.96	810.63 ± 20.68**	0.004
ACE	696.88 ± 25.23	796.64 ± 20.60*	0.010
Shannon	4.67 ± 0.09	5.01 ± 0.06**	0.006
Simpson	0.98 ± 0.003	0.99 ± 0.002*	0.033

^1^Data are expressed as mean ± SEM, n = 8 per treatment. ^*^P < 0.05 and ^**^P < 0.01 vs. the Control group. ICA100, the basal diet supplemented with 100 mg/kg indole-3-carboxaldehyde.

## Discussion

In this study, we evaluated the effects of dietary ICA supplementation on the growth performance, intestinal integrity, and gut microbiota composition in weaned piglets. We found that ICA did not alter ADG and ADFI, but significantly reduced F:G ratio in piglets after weaning. Moreover, ICA supplementation did not affect the intestinal morphology and permeability, and oxidative stress of piglets. Intriguingly, ICA treatment promoted intestinal index of piglets. *In vivo* and *in vitro* experiments further confirmed that ICA accelerated intestinal epithelial cell proliferation. In addition, dietary ICA supplementation changed the parameters and composition of colonic flora to some extent.

Due to early weaning, piglets face nutritional, environmental, physiological and other stress in advance, resulting in increased diarrhea, decreased digestive ability, and stunted growth (14). In the past, antibiotics were added to the feed to prevent diarrhea and improve growth performance in piglets (15). However, feed antibiotics have now been banned in many countries including China for their negative effects such as drug resistance. Therefore, the development of safe and green feed additives to replace antibiotics is of great significance. At present, plant extracts, acidifiers, enzymes, plant essential oils, probiotics, etc. are all options to replace antibiotics in feed. Besides, post-biotic metabolites are novel potential alternatives to antibiotics in animal feeding (16–18). In the current study, weaned piglets fed the diet supplemented with 100 mg/kg or 200 mg/kg ICA, a microbial tryptophan catabolite, did not affect ADG, ADFI, and diarrhea rate. Nevertheless, piglets supplemented 100 mg/kg ICA significantly decreased F:G.

Increasing studies have shown that microbial-derived metabolites modulate intestinal epithelial barrier. For example, short-chain fatty acids (SCFA: mainly acetate, propionate, and butyrate) are essential for enhancing intestinal barrier function and maintaining mucosal immunity. SCFA treatment upregulated mucin gene expression in intestinal epithelial goblet cells (19, 20). Butyrate reduced intestinal epithelial permeability and upregulated the expression of tight junction protein (5, 21). Microbial tryptophan catabolites also have been shown to enhance intestinal epithelial barrier, and exert an anti-inflammatory effect. Indole strengthened epithelial cell barrier properties by increasing tight junction resistance, and attenuated indicators of inflammation (6). Indole-3-propionic acid decreased FITC-dextran-dependent gut permeability in mice (22–24), increased mucin secretion in human colonic cells (9), increased goblet cell numbers and mucosa thickness in rats (25), and acted as a ligand of the aryl hydrocarbon receptor (AhR) and exerted an anti-inflammatory effect in colon (26). In this study, dietary ICA supplementation in weaned piglets did not alter intestinal morphological characteristics including the villus height, crypt depth, and villus height/crypt depth ratio, and had no influence on intestinal permeability as reflected by DAO, D-lactic acid and endotoxin concentration. These results differ from a previous study in a mouse model of colitis, which indicated that ICA protected against increased gut permeability, and maintained the integrity of the apical junctional complex (11).

Although ICA treatment had no effect on intestinal epithelial barrier function, it clearly promoted intestinal epithelial development. Dietary ICA supplementation significantly increased the jejunum and colon weight in pigs. Enhanced intestine development may be responsible for the improved feed conversion ratio by ICA supplementation. Furthermore, the expression of proliferation marker protein PCNA in the intestine was up-regulated by ICA treatment. There are several studies showing that other microbial metabolites modulate intestinal epithelial cell proliferation. For instance, butyrate stimulated proliferation of colonic cells or induced cell cycle arrest and apoptosis depending on its luminal concentration (27, 28). Gerard *et al*. showed that butyrate suppressed intestinal stem cell proliferation upon exposure (29). Lactate accelerated intestinal stem-cell-mediated epithelial development (4). In this study, *in vitro* experiments verified the promoting effect of ICA on intestinal epithelial cell proliferation. The expression of the ICA receptor AHR, and the proliferation markers PCNA and Cyclin D1 were all increased by 15 μM ICA treatment in IPEC-J2 cells. Whether ICA promotes intestinal epithelial proliferation through AHR signaling requires further study.

Some microbial tryptophan metabolites are suggested to exert anti-oxidative effect in systemic circulation (30). Wlodarska *et al*. found that indoleacrylic acid, but not indolepropionic acid, had an anti-oxidative and anti-inflammatory function in LPS-activated human peripheral blood mono-nuclear cells, which reduced IL-6 and IL-1β secretion, and activated the NRF2-ARE pathway (31). Indole-3-propionic acid protected cells from reactive oxygen species (ROS), oxidative damage, and lipid peroxidation (32–34). In the current study, dietary ICA supplementation in pigs did not change the antioxidant enzyme activities in serum, including SOD, GSH-Px, CAT, T-AOC, and MDA, suggesting ICA have no anti-oxidative effect in circulation.

Metabolites produced by gut bacteria, which in turn modulate gut microbiota composition. Zhao *et al*. (35) demonstrated that administration of indole-3-propionic acid modulated gut microbiota composition and inhibited microbial dysbiosis in rats fed a high-fat diet. Similarly, we found that dietary ICA supplementation resulted in a different microbial composition in the colon. The richness estimators and diversity index were both increased after ICA treatment. Besides, ICA supplementation decreased the abundance of phylum *Fibrobacterota*, altered the abundances of several genera including *Butyrivibrio, Alloprevotella, Prevotella*, and *Parabacteroides*. Taken together, the regulation of gut homeostasis by microbial metabolites includes their potential modulation of gut microbiota composition.

## Conclusion

Our results demonstrate that dietary ICA supplementation can reduce the feed to gain ratio of weaned piglets, which accomplished by accelerating intestinal epithelial proliferation without affecting intestinal morphology and permeability. In addition, ICA partially modulates the composition of intestinal flora. This study reveals a beneficial role for the microbial metabolite ICA on intestinal hemostasis in weaned piglets.

## Data Availability Statement

The datasets presented in this study can be found in online repositories. The names of the repository/repositories and accession number(s) can be found below: https://www.ncbi.nlm.nih.gov/, PRJNA815710.

## Ethics Statement

The animal study was reviewed and approved by Animal Care and Use Committee of Nanjing Agricultural University.

## Author Contributions

KY proposed the concept, contributed to the study design, wrote the manuscript, and participated in the entire research process. RZ participated in most of procedures, data acquisition and analyses, and drafted the manuscript. GH, YR, HW, YY, JG, and MW assisted in the experimental procedures. WZ was involved in the study design. All authors have read and approved the final manuscript.

## Funding

This study was funded by the National Natural Science Foundation of China (31972528), the Fundamental Research Funds for the Central Universities (KYGD202102), and the Jiangsu Agricultural Science and Technology Innovation Fund (CX(19)3012).

## Conflict of Interest

Author HW was a temporary employee of the project team. The remaining authors declare that the research was conducted in the absence of any commercial or financial relationships that could be construed as a potential conflict of interest.

## Publisher's Note

All claims expressed in this article are solely those of the authors and do not necessarily represent those of their affiliated organizations, or those of the publisher, the editors and the reviewers. Any product that may be evaluated in this article, or claim that may be made by its manufacturer, is not guaranteed or endorsed by the publisher.
